# Community based screening for sickle haemoglobin among pregnant women in Benue State, Nigeria: *I-Care-to-Know, a Healthy Beginning Initiative*

**DOI:** 10.1186/s12884-021-03974-4

**Published:** 2021-07-08

**Authors:** Osita U. Ezenwosu, Ijeoma U. Itanyi, Obiageli E. Nnodu, Amaka G. Ogidi, Fabian Mgbeahurike, Echezona E. Ezeanolue

**Affiliations:** 1 Centre for Translational and Implementation Research, University of Nigeria, Nsukka, Enugu Nigeria; 2 Department of Paediatrics, Faculty of Medical Sciences, College of Medicine, University of Nigeria, Nsukka, Enugu Nigeria; 3 Department of Community Medicine, Faculty of Medical Sciences, College of Medicine, University of Nigeria, Nsukka, Enugu Nigeria; 4 Centre of Excellence for Sickle Cell Disease Research & Training, University of Abuja, Abuja, Nigeria; 5 Caritas Nigeria, Federal Capital Territory, Abuja, Nigeria

**Keywords:** Sickle cell disease, Screening, Community-based, Awareness, Genotype

## Abstract

**Background:**

Haemoglobin genotype screening at prenatal care offers women an opportunity to be aware of their genotype, receive education on sickle cell disease (SCD) and may increase maternal demand for SCD newborn screening. In developed countries, most pregnant women who access prenatal care and deliver at the hospital receive haemoglobin genotype screening. In settings with low prenatal care attendance and low hospital deliveries, community-based screening may provide similar opportunity for pregnant women. We assessed the feasibility and acceptability of integrating haemoglobin genotype screening into an existing community-based HIV program.

**Methods:**

Onsite community-based integrated testing for HIV, hepatitis B virus and haemoglobin electrophoresis, were conducted for pregnant women and their male partners. Community Health Advisors implementing the NIH and PEPFAR-supported Healthy Beginning Initiative (HBI) program provided education on SCD, collected blood sample for haemoglobin electrophoresis and provided test results to participants enrolled into the HBI program. We concurrently conducted a cross-sectional study using a pretested, semi-structured, interviewer administered questionnaire to collect demographic data and assess awareness of individual haemoglobin “genotype” among HBI pregnant women participants.

**Results:**

In this study, 99.9% (10,167/10,168) of pregnant women who received education on SCD accepted and completed the survey, had blood drawn for haemoglobin electrophoresis and received their results. A majority of participating pregnant women (97.0%) were not aware of their haemoglobin “genotype”. Among the participants who were incorrect about their haemoglobin “genotype”, 41.1% (23/56) of women who reported their haemoglobin “genotype” as AA were actually AS. The odds of haemoglobin “genotype” awareness was higher among participants who were in younger age group, completed tertiary education, had less number of pregnancies, and attended antenatal care. Overall prevalence of sickle cell trait (AS) was 18.7%.

**Conclusions:**

It is feasible to integrate haemoglobin “genotype” testing into an existing community-based maternal-child program. Most pregnant women who were unaware of their haemoglobin “genotype” accepted and had haemoglobin genotype testing, and received their test results. Increasing parental awareness of their own haemoglobin “genotype” could increase their likelihood of accepting newborn screening for SCD.

**Supplementary Information:**

The online version contains supplementary material available at 10.1186/s12884-021-03974-4.

## Background

Sickle cell disease (SCD) is the most common haematologic genetic condition resulting from inheritance of two abnormal haemoglobin genes from both parents, leading to the development of abnormal sickle-shaped red blood cells with a variety of clinical manifestations [1–5]. Globally, SCD affects about 25 million people [6] and sub-Saharan Africa contributes approximately 75% of the global cases [4]. With an estimated SCD prevalence of 2% and a population of about 200 million, Nigeria has the largest proportion of individuals living with SCD worldwide [7–10]. Nigeria also contributes at least half of the global incidence of SCD with approximately 150,000 neonates born annually with this condition [8, 11, 12].

The risk factor for SCD is individuals who are heterozygous for sickle haemoglobin i.e. individuals with sickle cell trait (SCT) and there are over 40 million of these individuals in Nigeria including women [7–9]. Thus during pregnancy, women with SCT can deliver babies with SCD. If these babies with SCD are not identified early at birth through newborn screening and given timely basic interventions, they can manifest symptoms associated with the condition very early in life [10, 13, 14] suffer severe morbidity and early mortality with over 50% dying before 5 years of age [15, 16]. Those who survive beyond 5 years usually face multiple target organ complications and other morbidities which affect the quality of life and possible consequent mortality [17–24].

Despite the high incidence of SCD in Nigeria, newborn screening (NBS) for SCD is still not well established in Nigeria [25]. A recent feasibility study on implementing NBS as part of immunization program highlighted challenges in follow up of a substantial number of screen-detected babies as a limitation [26]. Also, parental unbelief of possibility of SCD in their apparently healthy newborns due to relative novelty nature of NBS in Nigeria has been noted [25]. Pregnancy, therefore, presents the opportunity to educate mothers on SCD, identify those who have SCT and educate them on NBS with emphasis on the need for follow up of screen-detected babies with SCD in order to reduce the complications associated with SCD when early interventions are initiated [27].

In developed countries, testing for haemoglobin genotype status among pregnant women is done in the hospital during prenatal care as part of comprehensive pregnancy tests [28]. However, in Nigeria most pregnant women miss being tested as only 54% of pregnant women access full antenatal care in health facilities [29]. Therefore, an innovative approach to increase the number of pregnant women screened for haemoglobin genotype outside the health facilities is needed. Researchers have identified integration of screening tests into existing community-based programs to provide a model that is culturally appropriate and acceptable [27, 30]. In our previous study, education and testing for Human immunodeficiency virus (HIV) were successfully integrated into existing Healthy Beginning Initiative (HBI) – a community-based health care programs for pregnant women in congregational setting supported by the President's Emergency Plan for AIDS Relief (PEPFAR) [31]. The HBI provides a culturally adaptive platform for screening, linkage and follow up of participants with the aim to respectively identify, treat and retain them in care [5, 32].

Accordingly, the aim of this study was to assess the feasibility and acceptability of integrating haemoglobin genotype screening into an existing community-based program for pregnant women and assess awareness of individual haemoglobin genotype among them. Increased haemoglobin genotype testing of pregnant women may increase the possibility of their demand for SCD screening of their newborns.

## Methods

### Study setting, design and participants

From June 2016 through October 2018, our study team sought to integrate screening for haemoglobin genotype into a community-based HBI program in Benue State, Nigeria supported by the United States National Institute of Health and Centers for Disease Control and Prevention. Benue State, north-central Nigeria has an estimated population of five million, most of the population live in rural areas and majority (70%) are peasant farmers [33]. The state has the second highest prevalence of HIV in Nigeria, at 4.9% [34]. A total of eighty churches in mostly rural communities across twelve LGAs in Benue State participated in the HBI. The HBI platform was utilized to consecutively recruit the pregnant women for assessment of the feasibility and acceptability of integrating haemoglobin genotype screening into the existing program.

A full description and effectiveness of the HBI, a community-based framework for maternal child health interventions has previously been described [5, 31, 32, 35]. In summary, HBI consists of three strategic components. 1) **Prayer sessions**: Each Sunday, the clergy leader announces for pregnant women and their male partners to come out for prayers. The clergy leader prays for a healthy pregnancy, safe delivery and encourages pregnant women to seek antenatal care at a health facility. The priest introduces the concept of the HBI and the study team. 2) Group **Baby showers** are organized as an integrated community reception and health fair in religious centers during which health education on early antenatal care (ANC), SCD, importance of the integrated screening tests for pregnant women, good nutrition, skilled birth attendance, and immunizations are provided by trained community health advisors. In addition to physical measurements (weight, height and blood pressure) taken, an onsite integrated laboratory testing for HIV, hepatitis B virus (HBV) and haemoglobin electrophoresis, are conducted for pregnant women and their partners. Male partners received a “mama pack” to present to their female partners as an expression of love and support during the pregnancy. Pregnant women and their male partners who wish to participate in a study are enrolled by the team after signing an informed consent and completing a baseline questionnaire. 3) **Baby receptions** are organized six to eight weeks after delivery, for women who participated in the baby shower. Participating women complete post-delivery questionnaire which provide an opportunity to ascertain place of delivery, pregnancy outcome and other related information. Enhanced nutrition and immunization education were provided. This also offered an opportunity for post-delivery linkage to care for women who needed care.

### Laboratory testing

After reviewing and confirming informed consent, trained phlebotomists collected 2 ml of whole blood from participants using sterile procedures. HIV and HBV rapid tests were performed onsite in an enclosed private location. The remaining blood sample was transported in cold boxes with ice packs to a central laboratory where haemoglobin electrophoresis was performed. This test identifies different haemoglobins by their migration within an electric field [7]. Haemoglobins variants move at different rates depending on their net negative charge which in turn is controlled by the composition (amino acids) of their hemoglobin molecule. Red blood cells were first lysed to release haemoglobin using water. The lysed samples were carefully applied on cellulose acetate paper alongside control samples and the test performed as per protocol in the National Guidelines [36]. The results were then read in comparison with control haemoglobin samples to identify the haemoglobin variants as AA, AS, AC, SS or SC.

### Questionnaire administration

We conducted a cross-sectional survey of HBI participants using questionnaires (Supplementary File) administered by trained research assistants who had a minimum of a Bachelor’s degree. The research assistants administered pretested semi-structured questionnaires during the baby showers individually to each pregnant woman and her male partner in a private location within the church premises. There were separate male and female questionnaires but only data from the female questionnaires are presented in this paper. Data was collected on sociodemographic characteristics and genotype awareness.

Sociodemographic characteristics included age, sex, marital status, highest level of education, occupation, monthly income, languages spoken, distance to health facility, and number of people living in the household.

### Ethical consideration

This study was approved by the Health Research Ethics Committee of the University of Nigeria Teaching Hospital, Enugu, Nigeria. Baby shower was a health fair for celebration of pregnancy and delivery of health education to pregnant women and their male partners. As such, consent was not needed to participate in the baby showers but written informed consent was obtained from the participants for laboratory tests and questionnaire data collection.

### Statistical analysis

Statistical analysis was performed using SPSS statistical program version 22 (IBM Corp Released 2013). The median and interquartile range were calculated for continuous variables while frequencies and proportions were generated for categorical variables. Chi-square test was used to test the association between variables in the study and genotype awareness. Bivariable and multivariable logistic regression analyses were used to determine the predictors of awareness of genotype status by estimating odds ratios and their 95% confidence intervals (CI) at a significance level of *p *< 0.05. Only variables which showed significant association in the bivariable analysis were included in the multivariable analysis.

## Results

### Characteristics of the study population

A total of 16,934 HBI participants including 10,168 pregnant women were recruited for the study out of which 97.9% (16,584) of all participants and 99.9% (10,167/10,168) of the pregnant women participants agreed to be screened for haemoglobin genotype after receiving education on SCD. Of the screened 10,167 women, their ages ranged from 13 to 59 years with overall median age of 24 years and interquartile range of 8. The sociodemographic characteristics of the participating women are shown in Table 1.

**Table 1:** Sociodemographic characteristics of the pregnant women participants of HBI in Benue State, Nigeria

Variables	*N* = 10,168
	**n (%)**
**Age in categories (years)**	
30	8,145 (81.1)
30 – 49	1,911 (18.8)
≥ 50	11 (0.1)
**Median age 24 years; IQR 8**	
**Marital Status**	
Single	24 (0.2)
Married	10,056 (98.9)
Widowed	67 (0.7)
Separated	15 (0.1)
Divorced	5 (0.0)
**Occupation**	
Unemployed	253 (2.53)
Civil servants	181 (1.8)
Farmers	8,835 (86.9)
Traders	632 (6.2)
Others	266 (2.6)
**Highest Educational level**	
No formal education	1,806 (17.8)
Primary	2,990 (29.4)
Secondary	4,720 (46.4)
Tertiary	651 (6.4)
**Income (Naira)**	
0 – 20,000	9,383 (92.3)
> 20,000 – 50,000	607 (6.0)
> 50,000 – 100,000	130 (1.3)
> 100,000	47 (0.5)
**Distance to facility (Km)**	
0 – 5 (Walk)	7,563 (74.4)
6 – 10 (Bike)	2,269 (22.3)
11 – 15 (Short ride)	234 (2.3)
> 15 (Long ride)	101 (1.0)

### Acceptability of haemoglobin genotype screening

Hundred percent of the recruited pregnant women agreed to participate in the haemoglobin genotype screening program and completed the semi-structured survey of their socio-demographic characteristics and genotype awareness. Ninety nine percent of them accepted to be screened for their haemoglobin genotype together with HIV and HBV testing. They also consented to have their blood sample taken for haemoglobin electrophoresis.

### Feasibility of integrating haemoglobin genotype screening into existing program

HBI provided a culturally-acceptable platform for the integration of haemoglobin genotype screening into an existing community-based screening program for HIV and HBV. During the integrated screening program, blood samples were successfully collected, transported to a central laboratory where they were screened for haemoglobin genotype and the results subsequently provided to all the tested pregnant women.

### Prevalence and awareness of haemoglobin genotype status

Table 2 shows results of haemoglobin genotype screening of the 10,167 pregnant women. Among these participants, 81.2% were found to have AA genotype, 18.7% were noted with AS while 5 (0.1%) had SS genotype. A vast majority (97%) of the pregnant women were not aware of their haemoglobin genotype status before the screening was conducted in this study. Among those who were incorrect about their haemoglobin genotype status, 41.1% (23/56) were actually AS although they had reported AA. (Table 3).

**Table 2:** Prevalence of different genotypes among pregnant women participants of HBI in Benue State, Nigeria

Genotype	Frequency	(%)
AA	8,252	(81.2%)
AC	3	(.0%)
AS	1,906	(18.7%)
SC	1	(.0%)
SS	5	(0.1%)
Total	10,167	(100%)

**Table 3:** Awareness of sickle haemoglobin status by pregnant women participants of HBI

	Genotype Awareness			Genotype Results			
	n	%	AA	AS	SS	AC	SC
Aware							
No	9,863	97.0					
Yes	304	3.0					
If Yes, Perceived genotype							
AA	238		**215**	23	0	0	0
AS	44		11	**33**	0	0	0
SS	19		13	6	0	0	0
AC	2		2	0	0	0	0
SC	1		1	0	0	0	0

### Factors associated with awareness of haemoglobin genotype status

Table 4 shows factors associated with awareness of haemoglobin genotype status among the pregnant women. Age, type of occupation, educational attainment, level of income, reception of antenatal care, number of pregnancies, and distance to health facility were associated with awareness of haemoglobin genotype status.

**Table 4:** Sociodemographic characteristics associated with awareness of sickle haemoglobin status among pregnant women participants

Genotype Awareness											
**Variables**	**Aware**		**(%)**		**Not Aware**	**(%)**		**χ**^**2**^		**df**	**p**
**Age in categories (years)**											
< 30	200		(2.4)		8,045	(97.6)		49.01		2	0.000
30 – 49	104		(5.4)		1,807	(94.6)					
≥ 50	0		(0.0)		11	(100)					
**Marital Status**											
Single		0	(0.0)		24	(100)		1.893		4	0.756
Married		303	(3.0)		9,753	(97.0)					
Widowed		1	(1.3)		66	(98.7)					
Separated		0	(0.0)		15	(100)					
Divorced		0	(0.0)		5	(100)					
**Occupation**											
Unemployed		44	(17.4)		209	(82.6)		790.94		4	0.000
Civil servants		41	(22.7)		140	(85.3)					
Farmers		108	(1.2)		8,727	(98.8)					
Traders		75	(11.9)		557	(88.1)					
Others		36	(13.5)		230	(86.5)					
**Highest Educational level**											
No formal education		16	(0.9)		1,790	(99.1)		621.48		3	0.000
Primary		33	(1.1)		2,957	(98.9)					
Secondary		133	(2.8)		4,587	(97.2)					
Tertiary		122	(18.7) 529			(81.3)					
**Income (Naira)**											
0 – 20,000		251	(2.7)	9,132		(97.3)	65.06		3		0.000
20 – 50,000		35	(5.8)	572		(94.2)					
50 – 100,000		17	(13.1)	113		(86.9)					
> 100,000		1	(2.1)	46		(97.9)					
**ANC**											
Yes		246	(4.4)	5359		(95.6)	84.24		2		0.000
No		58	(1.3)	4503		(98.7)					
**Gravidity**											
0 – 4		264	(3.2)	8096		(96.8)	6.84*		2		0.033
5 – 8		39	(2.3)	1633		(97.7)					
9- 13		1	(0.7)	134		(99.3)					
**Distance to facility (Km)**											
0 – 5 (Walk)		214	(2.8)	7,349		(97.2)	18.04		3		0.000
6 – 10 (Bike)		71	(3.1)	2,198		(96.9)					
11 – 15 (Short ride)		9	(3.8)	225		(96.2)					
> 15 (Long ride)		10	(9.9)	91		(90.1)					*Yates correction

The results of logistic regression analysis for predictors of haemoglobin genotype status awareness among the participants are shown in Table 5. Younger participants had higher odds of being aware of their haemoglobin genotype status compared to older participants. Compared to the unemployed participants, farmers had 5.8 times lower odds while the traders had 1.2 times higher odds of being aware of their haemoglobin genotype status. The odds of haemogobin genotype status awareness increased with increasing level of education, and increasing distance from the health facility. Also, the odds of a woman self-reporting her haemoglobin genotype status was 2 times higher among women who attended ANC compared to women who did not attend ANC.

**Table 5:** Predictors of sickle haemoglobin status awareness among pregnant women participants in Benue State, Nigeria

Variables	Adjusted Odds Ratio			
	AOR	*P*-value	95%	CI
**Age** (years)				
< 30	1			
30–49	1.821	< 0.001	1.359	2.439
≥ 50	0.000	0.999	0.000	
**Occupation**				
Unemployed	1			
Civil Servants	1.069	0.798	0.640	1.787
Farmers	0.171	< 0.001	0.110	0.268
Traders	1.249	0.339	0.792	1.971
Others	1.136	0.622	0.684	1.897
**Education**				
No Education	1			
Primary	1.128	0.697	0.615	2.068
Secondary	1.764	0.042	1.020	3.050
Tertiary	5.472	< 0.001	2.995	9.996
**Income** (Naira)				
0–20,000	1			
> 20–50,000	0.987	0.949	0.659	1.478
> 50–100,000	1.703	0.097	0.908	3.196
> 100,000	0.397	0.378	0.7051	3.106
**Gravidity**				
0–4	1			
5–8	0.855	0.437	0.575	1.270
9–13	0.358	0.317	0.048	2.676
**ANC**				
No	1			
Yes	2.084	< 0.001	1.535	2.829
**Distance** (Km)				
0–5	1			
6–10	1.199	0.225	0.894	1.608
11–15	2.147	0.043	1.024	4.502
> 15	3.935	0.001	1.758	8.808

## Discussion

Our study demonstrated that it is feasible to integrate screening for haemoglobin genotype into an existing community-based program. Overall, most pregnant women in the HBI lacked awareness of their own individual haemoglobin genotype status and accepted to be screened following informational sessions that provided education on sickle cell disease.

Other population-based studies have successfully integrated premarital screening for haemoglobin genotype into an existing health care system [37] and NBS into existing infectious disease and immunization programs [26, 38]. Our finding of very low awareness of individual haemoglobin genotype status among participants agreed with previous studies from Northern Nigeria, [39, 40] but was in contrast with high level of awareness found in Southern Nigeria. [41, 42] High acceptability of personal screening for haemoglobin genotype among the participants in this study was in keeping with earlier studies in Nigeria [39, 43] though poor participation in screening program has also been documented [44].

Integrating screening programs into established, culturally adapted and sustainable programs that meets the community needs have been shown to be key to successful program [45]. Most pregnant women participating in the HBI program lived in rural communities where access to health facilities was limited. The integrated approach to testing and integration of the screening program with a faith-based program that celebrates pregnancy, including the provision of “mama pack” which contains delivery room essentials was crucial to success of our program.

Increased awareness and knowledge of haemoglobin genotype status have been identified as important in strengthening public health efforts towards addressing SCD especially in high prevalence settings [46]. Higher level of education and effective health education are reported to impact on awareness of haemoglobin genotype status [47, 48]. The finding of very low awareness of personal haemoglobin genotype status among our participants and others from Northern Nigeria [39, 40] is not surprising as literacy level in the Northern Nigeria has been noted to be low compared to the South [49]. Also, limited access to health facilities in the North may explain the differences in the findings as health facilities are more in the South compared to the North [50] and health facilities serve as platform for dissemination of health education necessary for informed testing and awareness of haemoglobin genotype status.

In settings with limited facilities, inclusion of haemoglobin genotype screening campaign into existing health education program at the communities has been suggested [51]. Thus our study leveraged existing HBI implementing community health advisors to provide health education on SCD. Use of community health advisors who were highly respected and accepted by the community may explain the high level of acceptance for haemoglobin genotype screening among the participants.

In this large population-based study, the proportion of women with sickle cell trait was similar to the prevalence noted in other studies among pregnant women in Northeast, [52] Southeast [5] and Southwest [53] Nigeria. Though our figure fell within the prevalence of sickle cell trait in Nigeria which varied by geopolitical zones and ranged from 15–22%, it was higher than frequencies found in North Africa, [12] Southern Africa, [12] Europe and America [54]. The comparable frequency of sickle cell trait in our study could be due to the large sample size involved in this population-based testing as well as high uptake of screening as a result of the prior delivery of health education on sickle cell. The observed low prevalence of SCD in this and earlier studies [5] despite high annual incidence of SCD in newborns in Nigeria, [7] may be due to high morbidity of 50–80% in these newborns who rarely survive beyond 5 years thereby reducing the adult frequency [55].

### Strength of the study

The uniqueness of this study lies in its very large sample size and the community-based nature. It has been noted that majority of pregnant women in Nigeria do not attend prenatal care mainly because of distance to health facility [29]. Therefore a program like HBI that takes care and testing close to the pregnant women in their communities makes these services more accessible to them. This is important as antenatal care under-utilization is higher in the Northern compared to the Southern Nigeria [29]. To the best of our knowledge, this is the first study in the North Central Nigeria to integrate sickle cell screening with existing program for expectant mothers in an established infrastructure. Also, given the large sample size for the study, its findings are likely generalizable to pregnant women in the North Central zone of Nigeria.

### Limitation of the study

One major limitation of this study is the conduction of the haemoglobin genotype screening test in a rural setting with haemoglobin electrophoresis method which requires electricity to function. Though the samples for the test are transported in a cold box to a central laboratory for testing, the results are retrieved on subsequent days with possibility of problem with tracking the mothers who are seen again during baby receptions at 6 weeks post-delivery. This could be resolved with the use of point-of-care-test (POCT) for sickle cell which has high sensitivity and specificity compared to the gold standard- high performance liquid chromatography (HPLC) [56].

### Policy, clinical and research implications of our findings

The feasibility of integrating screening for haemoglobin genotype into an existing program as noted in this study shows that it is a beneficial alternative in a resource-limited setting and can be extended to other settings in Nigeria and sub-Saharan Africa.

In resource-limited settings, haemoglobin genotype screening of pregnant women can reduce the number of mothers whose newborns need to be screened for sickle cell disease. It will help identify women with SCT for targeted NBS of their infants which is a potentially useful public health strategy in resource-limited settings [5]. Thus for the viability of targeted newborn screening, all pregnant mothers need to be provided with screening for haemoglobin genotype to make them aware of their status. Subsequently, those with SCT can be identified so that only their at-risk newborns can be screened via targeted NBS [55]. This could be successfully implemented with the development and use of smartcard where the test results could be encrypted and read at the point of delivery using mHealth platform [57].

The low level of awareness of haemoglobin genotype status and high inaccurate report in our study points to the need for innovative interventions that will help increase community health education for increased testing acceptance and improved communication of test results. Following this, if more pregnant mothers are aware of their individual haemoglobin genotype status and are educated, they are more likely to demand for newborn screening of their babies.

## Conclusions

Our study is a large population-based study which demonstrated the feasibility of integrating screening for haemoglobin genotype into an established community-based screening program for pregnant women. It also showed that significant number of participants accepted screening following a tailored health education program. Increased acceptability of screening for haemoglobin genotype will increase individual awareness of haemoglobin genotype status among pregnant women. This could increase the likelihood of their demand for SCD NBS as well as follow up of screen detected infants for early interventions towards reduced morbidity and mortality.

## Supplementary Information

**Additional file 1**.

## Data Availability

The dataset generated and analyzed during this study is not publicly available due to confidentiality policies but are available from the corresponding author on reasonable request.

## Abbreviations

ANC: Antenatal Care
HBI: Healthy Beginning Initiative
HIV: Human Immunodeficieny Virus
NBS: Newborn Screening
NIH: National Institute of Health
PEPFAR: Presidents’s Emergency Plan For AIDS Relief
POCT: Point-Of-Care-Test
SCD: Sickle Cell Disease
SCT: Sickle Cell Trait
