# Supplementary material for: Community based screening for sickle haemoglobin among pregnant women in Benue State, Nigeria: I-Care-to-Know, a Healthy Beginning Initiative
Source: BMC Pregnancy Childbirth. 2021 Jul 8;21:498. doi: 10.1186/s12884-021-03974-4 (PMC8268197; doi:10.1186/s12884-021-03974-4)
Supplement: Supplementary file 1 — Additional file 1. [file 12884_2021_3974_MOESM1_ESM.pdf]

Member ID #

....

Bio Data Form

A2 - Completed at Baby Shower Registration

Site ID#........................

PARTICIPANT INFORMATION

1- Surname **2 - First Name** **3- Middle Name**

4 - Age **5 - Gender** ⑨ Male ⑨ Female

6 - Marital Status ⑨ Single ⑨ Married ⑨ Divorced ⑨ Separated ⑨ Widowed

Partner/Spouse's name:

7 - Descriptive Address

8 - Name of church you attend:

9 - Phone #1 **10 - Phone #2** **11 -Phone #3**

12 - Distance to closest health facility ⑨ 0-5 km *(walk)* ⑨ 6-10 km *(bike)* ⑨ 11-15 km *(short ride)* ⑨ >15 km *(long ride)*

13 - Occupation ⑨ Farmer ⑨ Trader ⑨ Civil Servant ⑨ Applicant ⑨ Other (specify)

14-Language(s) ⑨ Tiv ⑨ English ⑨ Igbo ⑨ Hausa ⑨ Yoruba ⑨ Other (specify)

15-Highest level of education ⑨ No education ⑨ Completed Primary School ⑨ Completed Junior Secondary ⑨ Completed Senior Secondary ⑨ Some Post-secondary ⑨ Completed Post- secondary

16 - Income per month ⑨ ₦0 - 20,000 ⑨ ₦20,001 - 50,000 ⑨ ₦50,001 - 100,000 ⑨ Above ₦100,001

17-How many other people are in your household? Adults: (over 18 years): Children (under 18):

Who do you want us to call if we can't find you? Remember, we will not share information with anyone other than you.

CONTACT PERSON 1

Surname **First Name**

Relationship to participant **Phone #1**

Phone #2

Descriptive Address

CONTACT PERSON 2

Surname **First Name**

Relationship to participant

Phone #1

Descriptive Address **Phone #2**

Date of Baby Shower..

Completed by...**Date** .... **Sign**..

Female Health Questionnaire

Completed at Baby Shower

Member ID # ....

Vital Signs Measurements	Height:	Weight:	Blood Pressure:

Medical History

1. Has a doctor ever told you that you have hypertension? ☐ YES ☐ NO
2. Has a doctor ever told you that you have diabetes? ☐ YES ☐ NO
3. Have you had any surgeries or operations or C-sections? ☐ YES ☐ NO
4. What is your genotype? ☐ AA ☐ AS ☐ AC ☐ SC ☐ SS ☐ Don't know
5. Have you ever been tested for HIV? ☐ YES ☐ NO

5a. When was your most recent HIV test? DATE: ☐ NA

5b. What was the result? ☐ NEGATIVE ☐ POSITIVE ☐ Not Sure Don't Know 5c. On HIV Drugs? ☐ YES ☐ NO

6. Have you ever been tested for Hepatitis B? ☐ YES ☐ NO

6a. When was your most recent Hepatitis B test? DATE: ☐ NA

6b. What was the result? ☐ NEGATIVE ☐ POSITIVE ☐ Not Sure Don't Know

Lifestyle Habits

7. How often do you drink alcohol? ☐ Never ☐ Daily ☐ Weekly ☐ Monthly ☐ Occasionally

7a. When was the last time you had a drink of alcohol?
☐ Yesterday ☐ Last 1 Week ☐ Last ______ Months ☐ More than a Year Ago ☐ N/A

8. How often do you use tobacco? ☐ Never ☐ Daily ☐ Weekly ☐ Monthly ☐ Occasionally

8a. When was the last time you used tobacco?
☐ Yesterday ☐ Last 1 Week ☐ Last ______ Months ☐ More than a Year Ago ☐ N/A

9. Do you use any other substance? ☐ YES ☐ NO
If yes, specify:

9a. How often do you use this substance? ☐ N/A ☐ Daily ☐ Weekly ☐ Monthly ☐ Occasionally

9b. When was the last time you used this substance?
☐ Yesterday ☐ Last 1 Week ☐ Last______ Months ☐ More than a Year Ago ☐ N/A

Reproductive Health

10. How many months is your pregnancy? ☐ 1 ☐ 2 ☐ 3 ☐ 4 ☐ 5 ☐ 6 ☐ 7 ☐ 8 ☐ 9 ☐ Don't know
11. How many times have you been pregnant before? ☐ 0 ☐ 1 ☐ 2 ☐ 3 ☐ 4 ☐ 5 ☐ 6 ☐ 7 ☐ 8 ☐ 9 ☐ >9
12. How many children do you have? MALE: FEMALE:
13. Are you receiving antenatal care? ☐ YES ☐ NO

13a. What is the name of the health facility? NAME:

14. What is your EDD (day or month)? DATE:
15. How many children do you wish to have? MALE: FEMALE:
16. Once you have a complete family, would you consider tying your womb? ☐ YES ☐ NO
17. Did you give breast milk to your last baby? ☐ YES ☐ NO ☐ N/A (1st Baby)
18. How do you plan to feed your baby for the first 6 months? ☐ Breast milk ☐ Water ☐ Formula ☐ Pap

Completed by:	Date:	Signature:
Reviewed by:	Date:	Signature:

Laboratory Results

Healthy Beginning Initiative

Date of test results: ....

Test	Result	Interpretation
Blood Pressure		
Hepatitis B		
HIV		
Sickle Cell Genotype		
Height		
Weight		

If you see a '+' in any row, one of your measures is abnormal and you should see a doctor. Bring this result to the doctor. If you do not understand your result or have any questions, contact your CHA.

Fold along line, staple and

Place Member ID Label on Outside of Form
